# 

**Published:** 1847-01

**Authors:** 


*Delegates to the National Convention.*—As many of the county societies of this State hold their annual meetings during the present month, we would remind members of the propriety of electing delegates to the National Convention at such meetings. We perceive that in several States representatives have been already chosen. We are gratified to state that the University of Pennsylvania, which, it had been supposed by some persons regarded the movement with indifference, has appointed delegates.

